# Association between age-related hearing loss and depression: A systematic review and meta-analysis

**DOI:** 10.1371/journal.pone.0298495

**Published:** 2025-01-03

**Authors:** Fuyao Li, Meiling Jin, Tianyi Ma, Chunlian Cui

**Affiliations:** Department of Otolaryngology Head and Neck Surgery, Yanbian University Affiliated Hospital, Yanji City, Yanbian Korean Autonomous Prefecture, Jilin Province, China; Universidad de Chile, CHILE

## Abstract

**Introduction:**

This meta-analysis examined the relationship between age-related hearing loss (ARHL) and depression in older adults, and further explored whether this relationship is moderated by age and gender.

**Methods:**

We searched in 4 English databases: PubMed, Embase, Web of Science, and Cochrane Library. Ultimately, we identified 9 studies, involving 3 cohort studies and 6 cross-sectional studies. We used Hedges’ g as the effect size, and all pooled analyses were performed using random-effects models.

**Results:**

ARHL patients had higher depressive symptom scores than non-ARHL older adults (g = 0.52). When divided into subgroups based on study type, a large effect size was demonstrated in the cross-sectional study group (g = 0.68) and was not statistically different in the cohort study group (g = 0.06). Meta-regression results showed that the effect size of depression in older adults with ARHL was significantly associated with the percentage of females (t = 5.97, p = 0.000) and not significantly associated with age (t = 0.94, p = 0.364).

**Conclusions:**

Patients with ARHL are more likely to be depressed than older adults with normal hearing, and this relationship is influenced by the gender of the patients.

## Introduction

Age-related hearing loss (ARHL), also known as presbycusis, is a complex disease caused by the cumulative effect of aging of the auditory system. It is defined as a progressive, bilateral, symmetrical multifactorial hearing sensitivity disease, which mainly occurs in the high-frequency region and is age-related sensorineural hearing loss [1]. ARHL is the third most common health condition affecting older adults, after heart disease and arthritis [2]. In addition, ARHL is the most common chronic sensory deficit in older adults and its prevalence increases dramatically with age. Its prevalence is estimated to be 25–30% in people aged 65–74 years [3], affecting 63.1% of people over 70 years, and 80.6% of people over 85 years [4]. In addition to the health burden, ARHL also brings huge economic costs. Studies have shown that in 2002 alone, the total cost of direct medical and productivity loss caused by hearing loss in adults aged ≥ 65 years in the United States was about 9.5 billion dollars. Considering the increase in life expectancy, it is estimated that the annual cost will increase to 60 billion dollars by 2030 [5]. Therefore, the health, social, and economic costs of ARHL are huge and increasing. ARHL has long been regarded as a benign result of aging, or ARHL will only affect the quality of life of older people, but relevant studies have shown that ARHL is associated with significant psychological and medical morbidity, including social isolation, weakness, depression, and cognitive decline [6–9]. This article will mainly discuss the relationship between ARHL (especially peripheral ARHL) and depression.

Cacciatore et al. first reported the relationship between hearing loss and depression in older adults, and concluded that older adults with hearing difficulties also had higher scores on the depression symptom scale [10]. This conclusion was further confirmed in a subsequent meta-analysis based on 7 studies and N = 17767 subjects [11]. In addition, further research shows that the speed of hearing loss in older adults is related to their social and emotional loneliness. If not treated in time, hearing loss may develop into a chronic stressor, leading to the spread of depression, which is called an additional stressor [12,13]. Therefore, hearing loss may aggravate the existing difficulties of older adults with psychosocial and functional abilities, and then increase the possibility of depression. In addition, some observational studies (cross-sectional and cohort studies) have also reported the association between ARHL and depression [14–17]. The above studies seem to indicate that there is a definite association between ARHL and depression in older adults. However, some subsequent studies denied this result, proving that there was no correlation between hearing loss and depression in the elderly [18–21]. Therefore, a meta-analysis is needed to integrate these different research results, to provide certain guiding significance for clinical practice.

Lawrence et al. did a meta-analysis on the relationship between hearing loss and depression in older adults in 2020 [22]. They included a total of 35 studies. Taking the OR value as the effect size, they statistically analyzed 147148 subjects and concluded that there was a statistically significant correlation between hearing loss and the incidence of depression in the elderly. Compared with Lawrence et al., who included all older adults who met the criteria of hearing loss, we have more stringent restrictions on them. Only older adults who met the criteria of age-related hearing loss were included. As a common type of hearing loss in the elderly, ARHL is also the most common chronic sensory defect in the elderly. It is more clinically meaningful to study the relationship between ARHL and depression in the elderly, which can provide more targeted psychotherapy and guidance for the elderly with hearing loss. In addition, a more meaningful innovation is that we use Hedges’ g as an effect size to evaluate the degree of depression in older people with ARHL compared with older people with normal hearing. Since the values obtained from the scale for evaluating depression in the elderly are generally continuous variables, it is more reasonable for us to use Hedges’ g as the effect size for the combined analysis of continuous variables.

This systematic review and meta-analysis mainly have the following two purposes: on the one hand, it is to explore whether there is a significant correlation between ARHL and depression in the elderly, so as to further verify the conclusion of Lawrence et al; On the other hand, we conducted regression analysis on the age and gender of the elderly, which also made up for the gap in the study of Lawrence et al., and has more guiding significance for clinical practice.

## Method

### Search strategy

This systematic review and meta-analysis followed the PRISMA statement [23] and is registered with PROSPERO (CRD42024496312). We searched the required literature through four English language databases: PubMed, Web of Science, Embase, and Cochrane Library. Online databases were searched to identify relevant articles from the first date of publication to December 13, 2023, the general search terms included the use of “age-related hearing loss”, and “depression”. For articles with missing or incomplete data, we will contact the authors of the article by email to obtain complete data. In addition, we will search the grey literature and then contact the authors in the field for relevant literature.

### Inclusion criteria

Studies were included if they (a) included community and/or high care facilities of subjects at least 55 years old with or without age-related hearing loss (ARHL), (b) used a cross-sectional or cohort design, (c)included measures of hearing loss (objective or subjective) and depression, and (d) provided depression scale scores at baseline for ARHL and non-ARHL.

Additionally, studies that met the following criteria were excluded: (a) the older adults included in the study only had hearing loss and did not meet the criteria for ARHL, (b) there is insufficient data in the article and the authors were unsuccessful in finding it, and (c) the included samples were not divided into hearing loss and non-hearing loss groups, or only older adults with hearing loss were included.

### Study selection

Two authors first independently read the title and abstract of the article for preliminary screening and then read the full text as well as data extraction and quality assessment. Any disagreements were resolved through discussion. At the stage of abstract reading, the kappa coefficient was 0.89. At the stage of full-text reading, the kappa coefficient was 0.88. Thus, the two authors showed substantial consistency in the process of literature screening.

### Quality assessment

The quality of included studies was assessed by two authors using the Newcastle Ottawa Scale (NOS) [24] for cohort studies or the Agency for Healthcare Research and Quality (AHRQ) [3] for cross-sectional studies, any disagreements were resolved through discussion. The NOS is a 9-point scale based on an 8-item checklist that consists of three domains: selection, comparability, and outcome. For each item in the checklist assessed, studies selected that were indicative of low risk of bias were awarded a star. Studies scoring 7–9 points, 4–6 points, and 3 or fewer points were at low, moderate, and high risk of biases, respectively. The AHRQ assessed six domains of bias (selection bias, performance bias, detection bias, attrition bias, reporting bias, and other bias). Based on the authors’ judgments, every article was rated as having a ‘low’, ‘high’, or ‘unclear’ risk of bias. In addition, we used funnel plots, and Egger’s test to count between-group publication bias for the primary outcome.

### Data extraction

Using a standardized form of data extraction, we extracted the following data: sample size, country, age, and sex of participants, type of study (cohort or cross-sectional study), measures of hearing and depression in older adults, and mean and SD of depression scales at baseline. For studies reporting subgroups of participants with dual sensory loss, only the hearing impairment subgroup was extracted. Corresponding authors were contacted when study information and necessary data to compute an effect size were not reported in published articles. To ensure the accuracy of the data, data were extracted independently by two authors located on separate occasions. Any disagreements were resolved through discussion.

### Statistical analysis

Data were analyzed using the Cochrane Review Manager (Review Manager Version 5.4) (Free software Downloaded from https://www.cochrane.org) and Stata 17.0 software. We used standardized mean difference (Hedges’ g) as effect size and calculated its 95% confidence interval (CI). In contrast to Cohen’s d, Hedges’ g weights the effect size by sample size, correcting for the tendency to overestimate in smaller studies (Noble et al., 2018). It is assumed that the effect size is small at g < 0.40, moderate at 0.40 < g < 0.70, and larger at g > 0.70. The significance level was set at P < 0.05.

First, we took all the studies as a whole and calculated the total effect size according to the depression symptom score at baseline, so as to evaluate the depression of older adults in the ARHL group and non-ARHL group. Second, we divided the included studies into cohort study groups and cross-sectional study groups according to the study type, and further analyzed the impact of study type on the results. Finally, we used meta-regression to analyze whether there was an association between depression and age and gender in older adults with hearing loss.

Considering the heterogeneity, all pooled analyses were performed using a random effects model. Cochrane’s Q and I^2^ statistics were used to test heterogeneity. If Q is statistically significant (P < .10), the I^2^ statistic estimates the percentage of sample differences due to heterogeneity. I^2^ values of 0%-40% (low), 41%-60% (medium), and 61%-100% (high) were used to classify the level of heterogeneity (Moher et al., 2009). In addition, we also performed a sensitivity analysis to examine whether deleting studies with abnormal characteristics could explain heterogeneity and affect the pooled effect.

## Results

**Fig 1:**
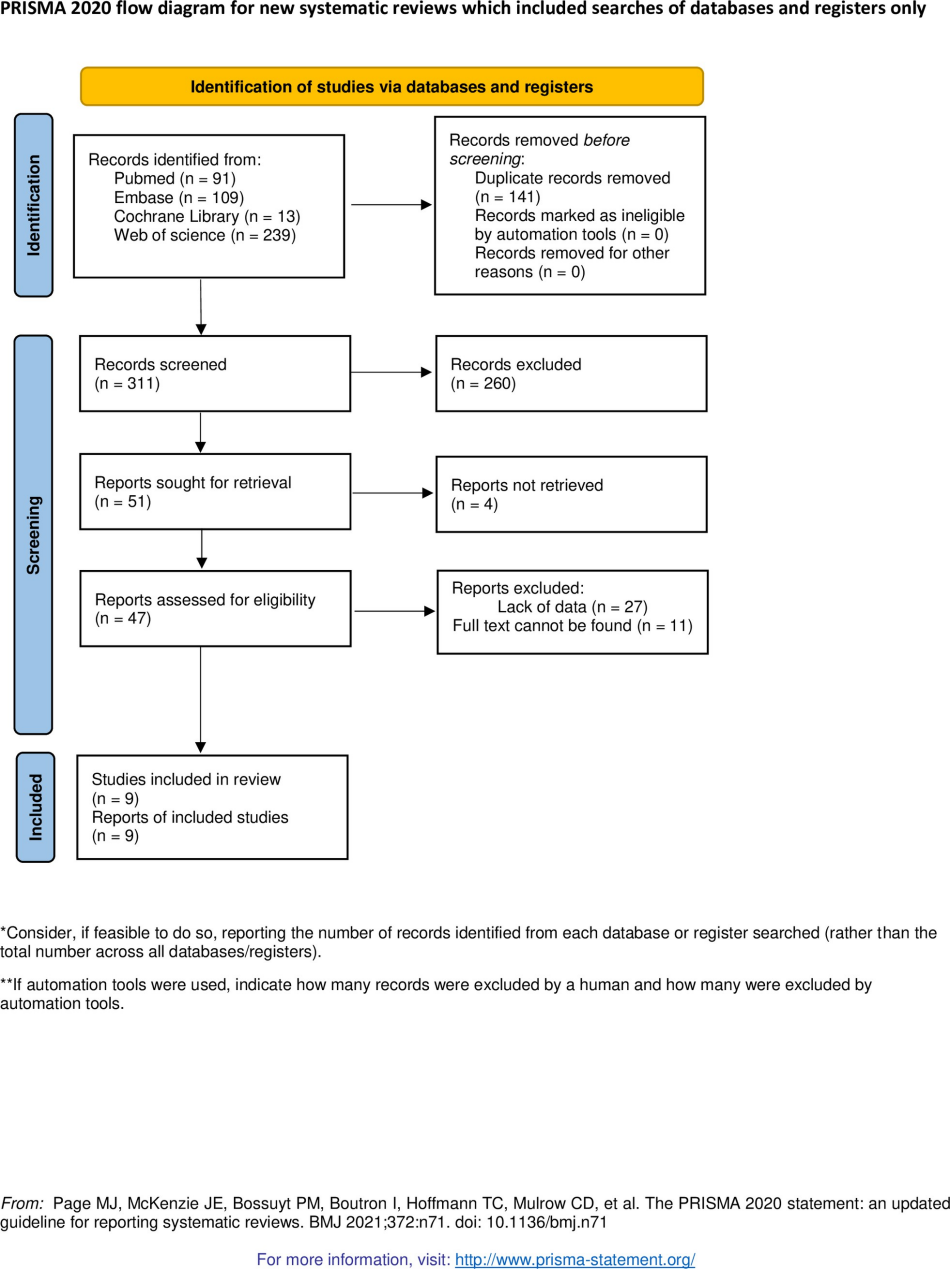
Literature screening process.

The preliminary search yielded 452 studies, 141 duplicate studies were deleted through the function of Endnote and then the remaining 311 studies were screened. By reading the title and abstract, we excluded 260 unrelated articles. Then the remaining 51 articles were full-text searched and read through, of which 4 were not retrieved, 27 were missing data, and 11 could not find the full text. Therefore, we finally included 9 studies for data extraction. The detailed process of screening is shown in Fig 1.

### Characteristics of the included studies

Table 1 provides details of each study. Nine studies included in this systematic review and meta-analysis were observational studies, including 3 cohort studies [21,25,26] and 6 cross-sectional studies [17,27–31]. A total of 25147 older adults were included in this meta-analysis, of whom 6046 had ARHL. The average age of the included samples was 69.02 years old, with the majority of women (62.77%). Six studies used objective measures of hearing loss and 3 studies used subjective measures. Nine studies have depression measurement scales and scores, such as 15 item Geriatric Depression Scale (GDS-15), Center for Epidemiological Studies Depression Scale (CES-D), the Depression Anxiety Stress Scales: DASS-21, and Hopkins Symptom Checklist (HSCL) In addition, 3 studies reported participants with cognitive decline.

**Table 1:** Characteristics of studies included in meta-analysis.

Study	Country	Study type	Sample number	Age (years)	Female (%)	Hearing assessment	Depression assessment	Cognitive ability assessment
Brewster 2021 [25]	United States	Cohort study	8529	73.90	63.45%	Clinical interview and physical examination	GDS-15	None
Brewster 2018 [27]	United States	Cross-section study	1194	73.10	53.09%	Self-reported	CESD-10	Modified MMSE
Cosh 2018 [21]	France	Cohort study	1467	73.34	49.50%	Self‐reported	HSCL	None
Feng 2022 [28]	China	Cross-section study	859	94.58	68.10%	Physical examination and hearing questionnaire	GDS-15	MMSE
Golub 2020 [30]	United States	Cross-section study	5499	62.20	61.50%	Pure-tone audiometry	CESD-10	None
Golub 2019 [29]	United States	Cross-section study	5328	58.50	61.60%	Pure-tone audiometry	CESD-10	None
Jayakody 2018 [31]	Australia	Cross-section study	151	64.44	51.66%	Pure-tone audiometry	DASS	None
Kiely 2013 [26]	Australia	Cohort study	894	78.00	49.20%	Pure-tone audiometry	CESD-10	None
Lee and Hong 2016 [17]	South Korea	Cross-section study	1226	75.77	100%	Self-reported	GDS-15	MMSE

Note: GDS-15, 15-item geriatric depression scale; CES-D-10, center for epidemiologic studies depression scale,10-item version; HSCL, hopkins symptom checklist; DASS, depression anxiety stress scales; MMSE, mini-mental state examination.

### Quality assessment and publication bias

**Fig 2:**
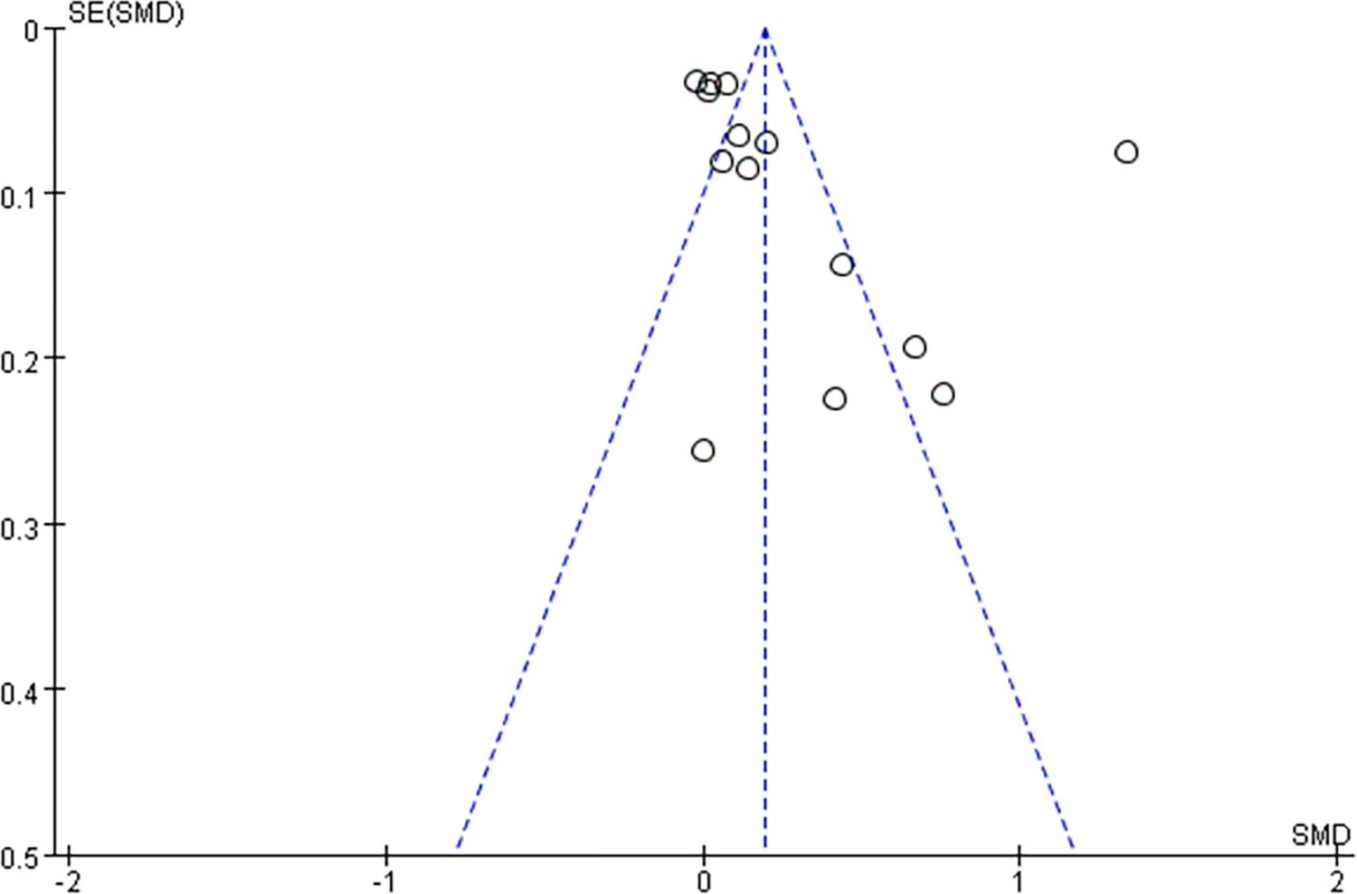
Relates the effect sizes of the studies to the standard errors, plotting a funnel plot for publication. SE, standard error; standardized mean difference (Hedges’ s g).

We included three cohort studies with NOS scores of 8, 8, and 7 (see Table 2), all of which were high-quality studies. We included 6 cross-sectional studies with AHRQ scores of 8, 9, 7, 8, 9, and 8 (see Table 3), all of which were high-quality studies. Therefore, the quality of the 9 studies we included is relatively high. In addition, we used the study’s effect size funnel plot and standard error (SE) to test for publication bias (see Fig 2). The subjective judgment did not show obvious publication bias, and then, we performed Egger’s test to quantitatively analyze it, so we further determined that there was no publication bias in this meta-analysis (t = 0.33, P = 0.7430).

**Table 2:** Quality assessment of included studies (Cohort studies).

COHORT STUDIES					
First author	Year	Selection	Comparability	Outcome	Overall quality score
Brewster	2021	★★★	★★★	★★	8
Cosh	2018	★★★	★★★	★★	8
Kiely	2013	★★★	★★	★★	7

Note: The Newcastle-Ottawa Quality Assessment Scale (NOS) was used to assess the quality of the included studies in three aspects, selection, comparison and results. The scores of cohort studies and case-control studies ranged from 0 to 9 and the higher the score, the higher the research quality. NOS scores ≥ 7, 4–6 and 0–3 represent high, medium and low quality, respectively.

**Table 3:** Quality assessment of included studies (Cross-sectional studies).

AUTHOR-YEAR	Brewster 2018	Feng 2022 [28]	Golub 2020 [30]	Golub 2019 [29]	Jayakody 2018 [31]	Lee and Hong 2016 [17]
Ⅰ	Y	Y	Y	Y	Y	Y
Ⅱ	Y	Y	Y	Y	Y	Y
Ⅲ	Y	Y	U	U	Y	Y
Ⅳ	U	U	U	Y	Y	U
Ⅴ	N	N	N	N	N	N
Ⅵ	Y	Y	Y	Y	Y	Y
Ⅶ	Y	Y	Y	Y	Y	Y
Ⅷ	N	Y	U	U	U	U
Ⅸ	Y	Y	Y	Y	Y	Y
Ⅹ	Y	Y	Y	Y	Y	Y
Ⅺ	Y	Y	Y	Y	Y	Y

Note: Y: Yes N: no U:Unclear Ⅰ: Was follow-up long enough for outcomes to occur Ⅱ: Is the case definition adequate Ⅲ: Same method of ascertainment for cases and controls Ⅳ: Define the source of information (survey, record review) Ⅴ: List inclusion and exclusion criteria for exposed and unexposed subjects (cases and controls) or refer to previous publi.cations Ⅵ: Indicate whether or not subjects were consecutive if not population-based Ⅶ: Explain any patient exclusions from analysis Ⅷ: Describe how confounding was assessed and/or controlled Ⅸ: Summarize patient response rates and completeness of data collection Ⅹ: Clarify what follow-up, if any, was expected Ⅺ: Clarify the percentage of patients for which incomplete data or follow-up was obtained

### Age-related hearing loss and depression

**Fig 3:**
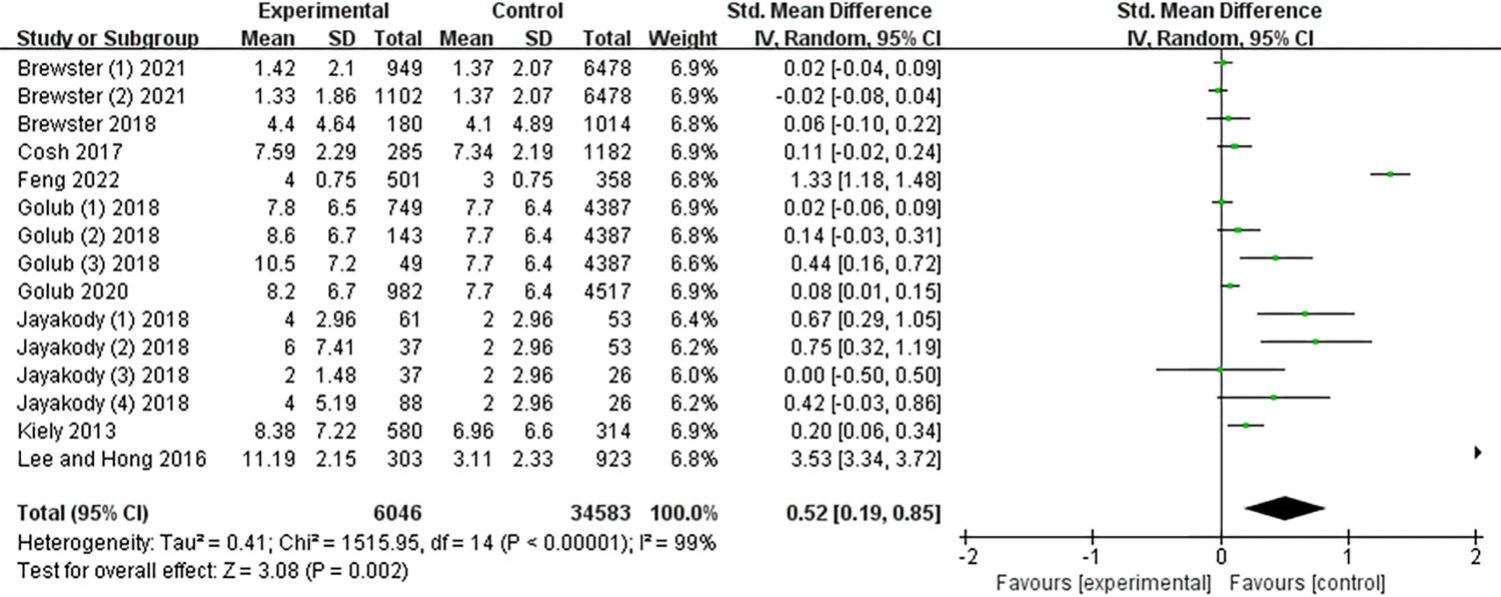
Meta-analysis of the relationship between ARHL and depression in older adults.

Older adults with ARHL were more likely to be depressed compared to those with normal hearing (g = 0.52; 95%CI: 0.19 to 0.85; p < 0.05; I^2^ = 99%; see Fig 3). There was a high degree of heterogeneity among these studies. Exploring the source of heterogeneity through sensitivity analysis revealed that three studies, Feng et al. (2022), Jayakody et al. (2018), and Lee and Hong. (2016), may be responsible for the high heterogeneity of the meta-analysis [17,28,31]. When these studies were excluded, I^2^ decreased from 99% to 61%, showing moderate heterogeneity.

### Study type for subgroup analysis

**Fig 4:**
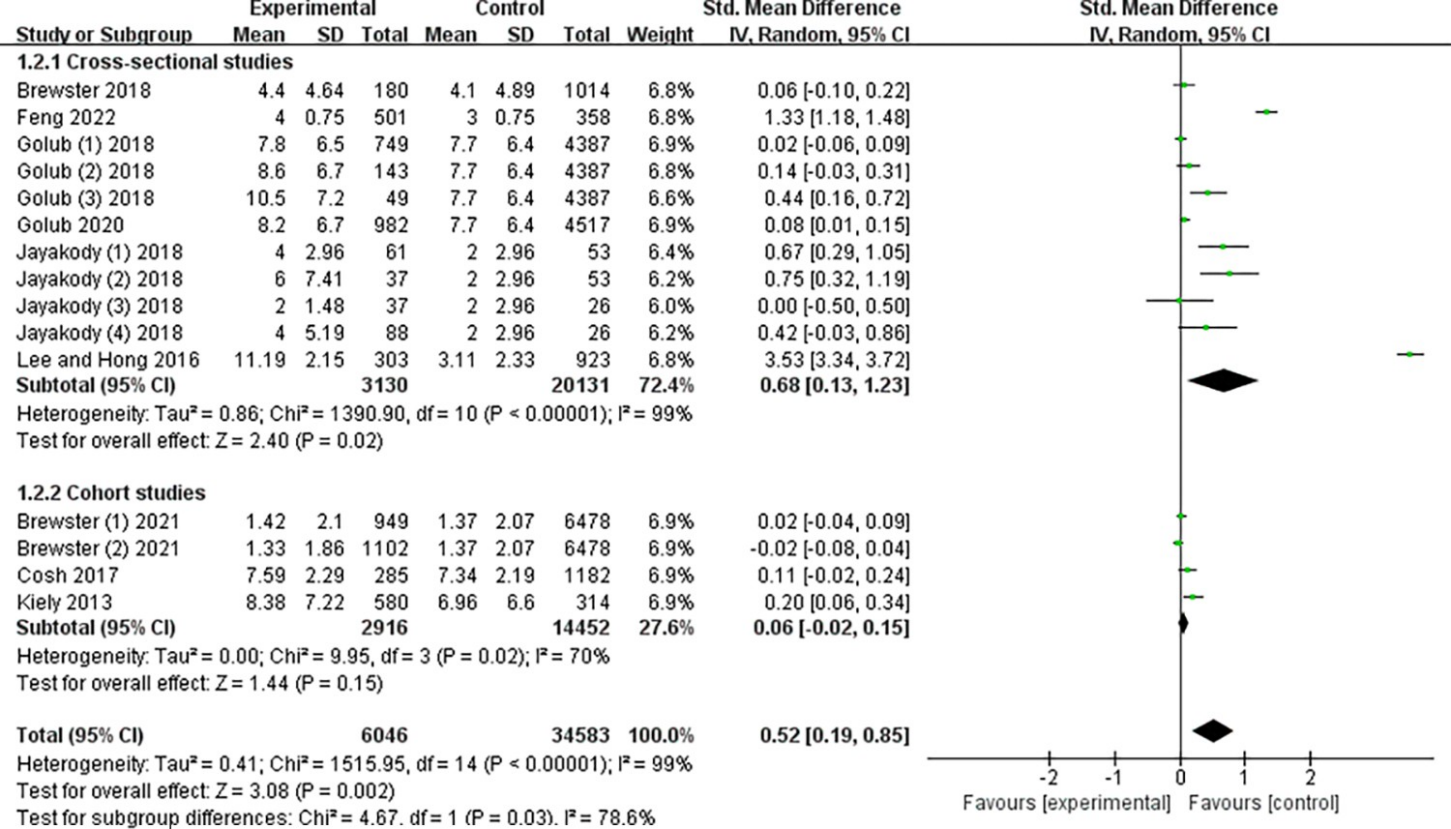
Meta-analysis by subgroups according to study type.

We divided the included studies into two subgroups according to the type of study: the cohort study group and the cross-sectional study group. In the cross-sectional study group, older adults with ARHL were more likely to be depressed compared to those with normal hearing (g = 0.68; 95%CI: 0.13 to 1.23; p < 0.05; I^2^ = 99%; see Fig 4). In the cohort study group, older adults with ARHL did not show a statistical difference in developing depression from older adults with normal hearing (g = 0.06; 95%CI: -0.02 to 0.15; p < 0.05; I^2^ = 70%; see Fig 4).

### Hearing assessment type for subgroup analysis

**Fig 5:**
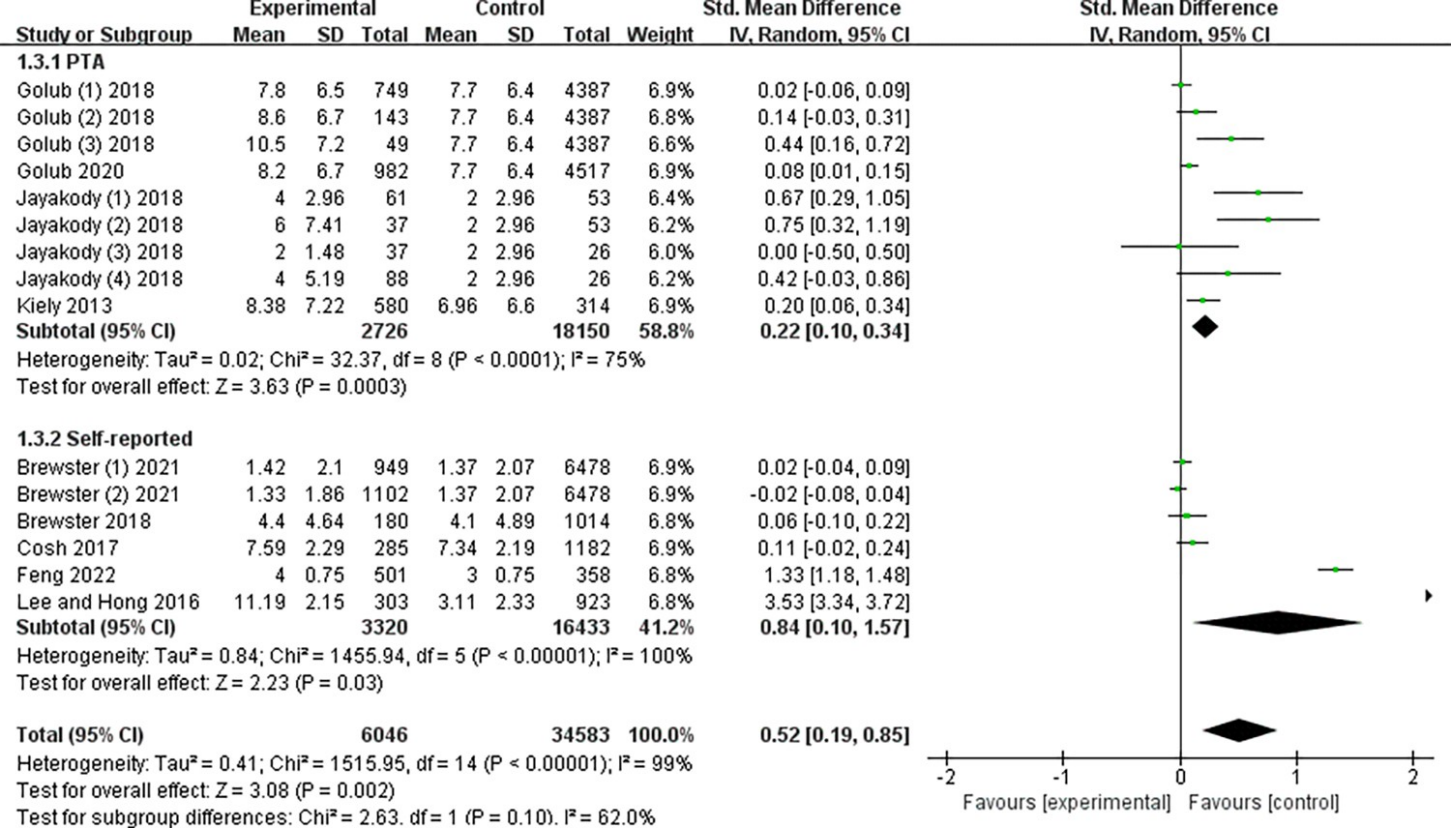
Meta-analysis by subgroups according to type of hearing assessment.

Then, according to the type of hearing assessment, we divided the included studies into two subgroups, the PTA group and the SR group. In the PTA group, older adults with ARHL were more likely to be depressed compared to those with normal hearing and showed a small effect size (g = 0.22; 95%CI: 0.10 to 0.34; p < 0.05; I2 = 75%; see Fig 5). In the SR group, older adults with ARHL were more likely to be depressed compared to those with normal hearing and showed a large effect size (g = 0.84; 95%CI: 0.10 to 1.57; p < 0.05; I2 = 75%; see Fig 5).

### Effect size as a function of age and percentage of females

Meta-regression results showed that the effect size of depression in older adults with ARHL was significantly associated with the percentage of females (t = 5.97, p = 0.000) and not significantly associated with age (t = 0.94, p = 0.364).

## Discussion

In this systematic review and meta-analysis, we mainly studied the relationship between age-related hearing loss (ARHL) and depression in older adults, quantified this result by calculating the score of the depression scale, and reached meaningful conclusions. A similar meta-analysis was done by Lawrence et al. (2020), which differed from ours in that they did not qualify subjects, and older adults who qualified as having hearing loss were included [22]. Therefore, all types of hearing loss were included in their study. However, Lawrence et al. (2020) did not divide hearing loss into subgroups according to types when conducting a synthetic analysis of these included studies, but analyzed the relationship between all types of hearing loss and depression as a whole [22]. Although this conclusion provides some ideas for clinical diagnosis and treatment, its practical application in clinics is not so extensive. As we know, when diagnosing a hearing loss patient clinically, it is usually classified according to its symptoms and examination results, such as conductive hearing loss, sensorineural hearing loss, and mixed hearing loss [32]. Therefore, we are often more interested in the relationship between a specific type of hearing loss and depression in older adults. ARHL patients included in our review are a type of sensorineural hearing loss. As the most common chronic sensory defect in the elderly, the prevalence of ARHL increases sharply with age. Therefore, it is of broad clinical significance to study the relationship between ARHL and depression in older adults.

Our quantitative analysis of 9 studies, N = 25,147 subjects, yielded 3 meaningful results: first, we concluded that there is a definite relationship between ARHL and depression in older adults when we took all the studies as a whole, which further confirms the conclusions of Lawrence et al. (2020) [22]. Since depression scale scores are continuous variables, it is more reasonable for us to choose Hedges’ g as an effect indicator as opposed to Lawrence et al.’s (2020) use of OR values as an effect size. By extracting the mean and standard deviation of depression scale scores at baseline for both ARHL patients and non-ARHL, and conducting quantitative synthesis and analysis, it was concluded that older adults with ARHL had higher depression scale scores than non-ARHL and demonstrated a moderate effect size (g = 0.52). Both Contrera et al. (2017) and Pronk et al. (2014) showed that older adults with hearing loss may experience emotional and social loneliness [12,33]. Jayakody et al. (2018) and Gopinath et al. (2012) showed that older adults with hearing loss have poor cognitive function and difficulties in completing daily activities [34,35]. These are independent risk factors for depression in older adults [36,37]. Therefore, hearing loss may increase the risk associated with psychosocial and functional abilities in older adults, thereby increasing the likelihood of depression. Within the stress process paradigm, the degree of social support can explain the relationship between hearing loss and depression in older adults. West et al. (2017) found in a large longitudinal study of American adults that without sufficient social support, hearing loss can become a chronic stressor of older adults, which in turn leads to the spread of depression [13]. These studies have confirmed the accuracy of our conclusions. Therefore, for the elderly with ARHL, we should pay special attention to their mental health, and carry out psychological intervention for the elderly with depression as soon as possible to prevent their depressive symptoms from further aggravating.

Secondly, we divided the included studies into two subgroups, the cohort study group, and the cross-sectional study group, and concluded that in the cross-sectional study group, the depression score of ARHL patients was higher and showed a large effect size (g = 0.68), but there was no significant difference in the cohort study group, which was contrary to the conclusion of Lawrence et al. (2020) [22]. The main reason for this result is that we included relatively few studies in the cohort study group, only three, which led to the quantitative synthesis results being greatly affected by a single study, thus affecting the accuracy of the results. At the same time, we should also consider the risk that the results of cohort studies may not be so accurate, which needs further confirmation by subsequent scholars who include more cohort studies.

Finally, we conducted a meta-regression analysis on the age of the subjects and the percentage of women, which filled the gap in the study of Lawrence et al. (2020) [22]. The results of the regression analysis for age showed that the age of the subjects did not affect the effect size (p = 0.364), a result that is not very plausible. Related studies have shown that age is an independent risk factor for ARHL and that the prevalence of ARHL in older adults increases sharply with age [9]. In addition, age is simultaneously a risk factor for depression in older adults, and the prevalence of depression in older adults continues to increase with age [38]. Therefore, we can speculate that age is a moderator of depressive symptoms in ARHL patients, which needs further confirmation by subsequent studies. The results of the regression analysis for gender showed that the percentage of females affected the effect size (p = 0.000), which also provides a new research idea that researchers in related fields may consider dividing older adults with ARHL into two subgroups of males and females, which in turn will explore the differences in concurrent comorbid depression between male and female ARHL patients.

Through reading a large number of literature in this field, we found some relatively novel research directions, but due to the impact of the number of included studies, we could not conduct a combined analysis of them, hoping to provide some ideas for researchers in this field. In the studies of Jayakody et al. (2018) and Golub et al. (2019), older adults with hearing loss were categorized according to severity as mild hearing loss, moderate hearing loss, and moderately severe or worse hearing loss, and concluded that there were differences in depressive symptoms among older adults with different degrees of hearing loss [29,31]. In addition, studies by Cosh et al. (2018) and Kiely et al. (2013), on the other hand, included patients with both dual sensory impairments (visual and auditory impairments) and concluded that older adults with dual sensory impairments may be more susceptible to depression than older adults with hearing loss alone [21,26]. In addition, three of the 9 studies we included described cognitive impairment associated with ARHL [17,27,28]. However, we did not analyze them synthetically due to the small amount of data, which is a regret of our study.

Our systematic review and meta-analysis obtained some meaningful results, and to some extent, made up for the gap in the study of Lawrence et al. (2020), but our article still had some shortcomings: (a) we included relatively few studies (only 9 studies), which led to the relatively poor reliability of the results obtained when we carried out quantitative synthesis analysis, and even false-positive results may occur [39]. Therefore, it is hoped that future researchers can further verify our research results by a more detailed search, including more studies and a larger sample size when conducting relevant meta-analysis; (b) 9 studies we included are observational, lack of randomization, and there are often differences between different groups at baseline. The quality of the articles is not as good as randomized controlled trials (RCTs), so it may lead to a certain bias in the results of quantitative synthesis. Since scholars in this field do cohort studies or cross-sectional studies without RCTs, this risk of bias is inevitable. Lawrence et al. (2020) also face such a problem; (c) after quantitative synthesis of all included studies, we found that there was a high heterogeneity (I^2^ = 99%), which may be caused by differences in experimental design between studies. After we divided the included studies into cross-sectional study group (I^2^ = 99%) and cohort study group (I^2^ = 70%) according to the study type, we found that the heterogeneity of the cross-sectional study group was still high, while that of the cohort study group was reduced. In addition, through sensitivity analysis, we concluded that the heterogeneity of Feng (2022), Jayakody et al. (2018), and Lee and Hong (2016) had significantly decreased (I^2^ = 61%) after the three studies were removed. These three studies are all cross-sectional studies, so we speculate that the differences in study design (randomization, allocation concealment, blinding, etc.) between studies (especially cross-sectional studies) lead to the high heterogeneity of articles. It is hoped that future scholars can include studies with similar experimental designs for synthetic analysis to make up for our shortcomings in this regard.

## Conclusion

This systematic review and meta-analysis had two main findings. On the one hand, we confirmed that there is a definite relationship between ARHL and depression in older adults, which further confirms previous findings. On the other hand, we found that gender was a moderator of the effect size; unfortunately, we did not find an effect of age on this relationship, both of which need to be further confirmed by future studies. Based on our conclusions from this meta-analysis, we suggest that we should focus on the mental health status of older adults with ARHL, and intervene in the treatment of older patients who already have comorbid depression to prevent further deterioration of the condition.

## Supporting information

S1 Checklist PRISMA checklist. (DOCX)
